# Artificial intelligence: A new tool in the pathologist's armamentarium for the diagnosis of IBD

**DOI:** 10.1016/j.csbj.2024.09.003

**Published:** 2024-09-11

**Authors:** Anna Lucia Cannarozzi, Luca Massimino, Anna Latiano, Tommaso Lorenzo Parigi, Francesco Giuliani, Fabrizio Bossa, Anna Laura Di Brina, Federica Ungaro, Giuseppe Biscaglia, Silvio Danese, Francesco Perri, Orazio Palmieri

**Affiliations:** a Division of Gastroenterology, Fondazione IRCCS - Casa Sollievo della Sofferenza, San Giovanni Rotondo, Italy; b Gastroenterology and Digestive Endoscopy Department, IRCCS Ospedale San Raffaele, Milan, Italy; c Innovation & Research Unit, Fondazione IRCCS "Casa Sollievo della Sofferenza", San Giovanni Rotondo, Italy; d Faculty of Medicine, Università Vita-Salute San Raffaele, Milan, Italy

**Keywords:** IBD, CD, UC, Histological analysis, AI

## Abstract

Inflammatory bowel diseases (IBD) are classified into two entities, namely Crohn's disease (CD) and ulcerative colitis (UC), which differ in disease trajectories, genetics, epidemiological, clinical, endoscopic, and histopathological aspects. As no single golden standard modality for diagnosing IBD exists, the differential diagnosis among UC, CD, and non-IBD involves a multidisciplinary approach, considering professional groups that include gastroenterologists, endoscopists, radiologists, and pathologists. In this context, histological examination of endoscopic or surgical specimens plays a fundamental role. Nevertheless, in differentiating IBD from non-IBD colitis, the histopathological evaluation of the morphological lesions is limited by sampling and subjective human judgment, leading to potential diagnostic discrepancies. To overcome these limitations, artificial intelligence (AI) techniques are emerging to enable automated analysis of medical images with advantages in accuracy, precision, and speed of investigation, increasing interest in the histological analysis of gastrointestinal inflammation. This review aims to provide an overview of the most recent knowledge and advances in AI methods, summarizing its applications in the histopathological analysis of endoscopic biopsies from IBD patients, and discussing its strengths and limitations in daily clinical practice.

## Graphical Abstract

**Figure:**
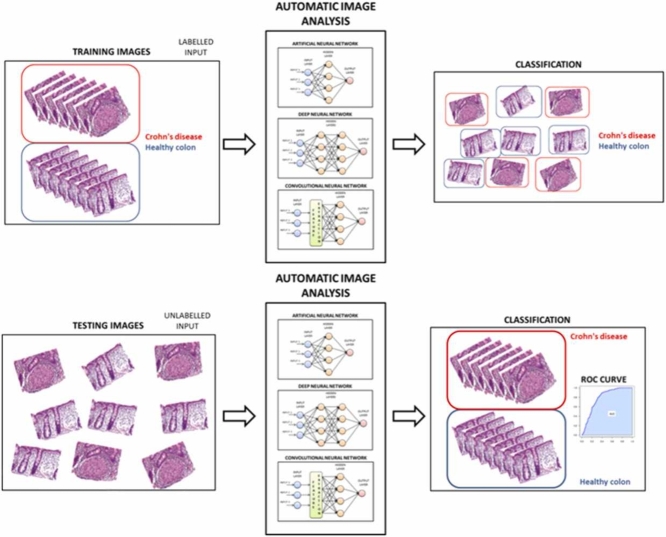

## 1 Introduction

Inflammatory bowel diseases (IBD) are chronic disorders that can affect any segment of the gastrointestinal tract and are classified into two entities: Crohn's disease (CD) [1] and ulcerative colitis (UC) [2]. These entities differ in genetic, epidemiological, clinical, endoscopic, and histopathological aspects, as well as in disease course [1], [2]. An accurate differential diagnosis of IBD is based on clinical features, endoscopy, histopathology, imaging, and laboratory tests [3]. Traditionally, the severity of IBD is classified into four groups: remission, mild, moderate, and severe activity, which are assessed using several structured tools [4], [5]. Besides, the disease location is defined according to the Montreal classification [6]. As there is no single golden standard modality for the diagnosis of IBD, the diagnostic process requires a multidisciplinary approach, including gastroenterologists, endoscopists, radiologists, and pathologists. Histological examination of endoscopic biopsies or surgical specimens plays a fundamental role in the differential diagnosis between IBD and non-IBD colitis. To ensure accurate histological evaluation of clinical suspicion of IBD, it is strongly recommended that all segments of the ileocolonic tract be sampled for histological examination during endoscopy. The diagnosis and differential diagnosis of IBD is based on the evaluation of the “minimal histological lesions” present in each of the different colic tracts: changes in the glandular component (glandular loss or atrophy; glandular architectural morphological changes or distortion); changes in the cellular component of the glands (mucodepletion; muciparous hyperplasia; Paneth cell metaplasia in the left colic segments, sigma and rectum; pseudopiloric metaplasia in the terminal ileum); the composition of the inflammatory infiltrate in the lamina propria (lymphoplasmacytic and granulocytic, with or without eosinophils) and its distribution in the segments examined (homogeneous vs heterogeneous). Other minimal lesions, which are not always detectable, include erosion/ulceration and non-necrotizing microgranulomas. The presence of granulocytes within the glandular epithelium (cryptitis) and/or within the lumen of the glands (cryptic pseudo abscesses) identifies disease in the active phase. Some minimal lesions, such as morphological changes in the glands and lymphomonocytic infiltration of the lamina propria, require grading into mild (lesions present in <30 % of the mucosa), moderate (lesions present in 30 % to 60 % of the mucosa) and severe (lesions present in >60 % of the mucosa) [7]. However, histopathological evaluation of morphological lesions to differentiate IBD from non-IBD colitis, although conducted by experienced and qualified pathologists, is limited by the high workload required and inter-observer variability. Furthermore, there is no consensus on the definition of each of the “minimal lesions” and their respective thresholds, as stated in the European Crohn’s and Colitis Organization position paper [8]. Morphological evaluation is influenced by subjective human judgment, leading to potential diagnostic discrepancies. [9]. Currently, in settings that require the highest reliability, such as clinical trials, centralized reading of histological biopsies is used to reduce interobserver variability. However, this approach does not eliminate subjectivity and is expensive and time-consuming. To overcome these limitations, the application of emerging computer-aided techniques and methods, commonly referred to as "artificial intelligence" (AI), which allows automated analysis of histological images, should be investigated as a novel way to achieve more reproducible and standardized diagnoses. [10]. Although computational methods and AI algorithms are becoming increasingly popular in the management of patients with IBD [11], evidence for their application in clinical practice and histology is still lacking. Imaging techniques applied to pathologies other than IBD, particularly in the field of oncology [12], have brought numerous advantages in terms of accuracy, precision, and speed of examination, generating interest in the histological analysis of gastrointestinal inflammation [13]. The hypothesis of generating multi-modal predictive clinical tools through the integration of “endo-histo-omics” data is also gaining strength, which could provide a broader and more comprehensive understanding of the disease, thus transforming the assessment and management of IBD patients and creating new approaches to patient care [14].

This review aims to describe the latest findings and advances in AI methods, summarize their applications in the histopathological analysis of endoscopic biopsies from IBD patients, and discuss their strengths and limitations in daily clinical practice.

## 2 Methods

The narrative review was conducted using the free PubMed database from 2013 to 2024, employing MeSH terms related to IBD, such as "inflammatory bowel disease," "ulcerative colitis," and "Crohn’s disease," separated by the Boolean operator "OR," and combined with the Boolean operator "AND" with MeSH terms related to AI tools, such as "artificial intelligence," "machine learning," "deep learning," "artificial neural networks," "convolutional neural networks," "computer-aided systems," and "automated image analysis." Studies on AI applications not related to image analysis and their classification in the field of IBD were excluded from this search. Additional MeSH terms, such as "diagnosis," "classification," "histology," "histopathological evaluation," and "pathology" were included to obtain only studies related to automated histopathological image analysis on IBD. There were no restrictions on the number of subjects and type of AI strategy for the included studies.

### 2.1 Artificial intelligence concepts

**Fig. 1:**
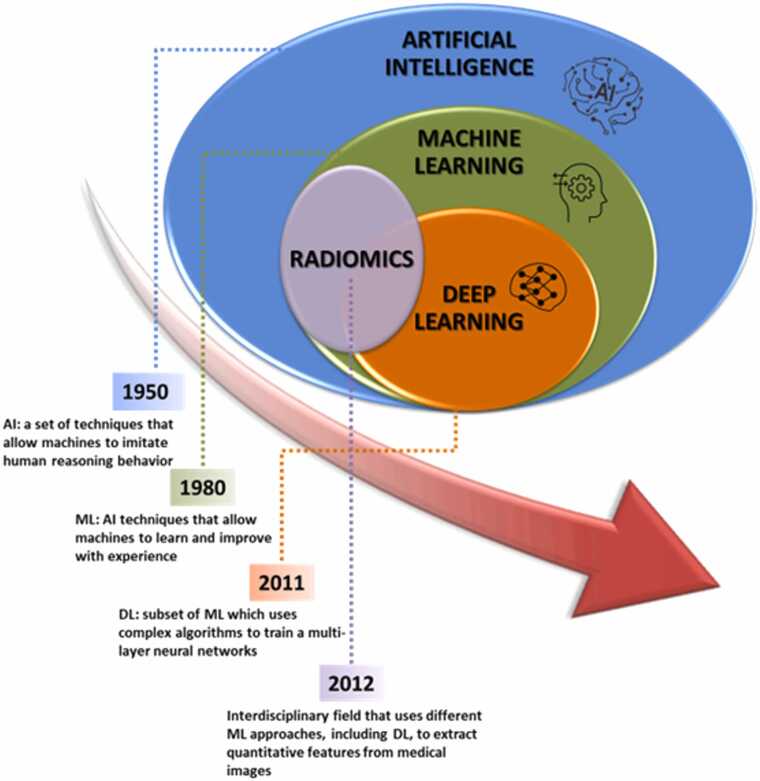
Graphical representation of elementary concepts linked to artificial intelligence (AI) and their historical appearances. ML: machine learning; DL: deep learning.

AI is a broad and multidisciplinary field encompassing specific subsets, namely machine learning (ML), deep learning (DL), and radiomics [15] (Fig. 1). It aims to develop systems capable of performing tasks and solving problems that require human intelligence and decision-making [16].

Moreover, AI is a powerful tool for analyzing big data and studying complex diseases, on problems that traditional tools struggle to resolve [17]. Using sophisticated and elaborated computational algorithms based on pattern extraction, AI analyzes significant electronically stored data and provides predictive results. These results can support human judgment, helping expert clinicians in decision-making processes and the discovery of new features underlying multifactorial diseases.

Machine learning is an AI approach widely used to solve problems by adopting a human-like learning process that improves through exposure to new data. In general, an ML model is built using a training dataset, processed through analytical computational techniques, and capable of estimating the probability of a possible outcome. The training model can be developed in different ways [18]: supervised learning [19], using human-labeled input-output pairs in the training dataset to determine outputs for unlabeled inputs; unsupervised learning [20], characterized by the absence of labeled input data; reinforcement learning [21], a process in which decision-making is directed toward achieving a given goal through interactions with the environment.

Deep learning is a subset of ML where information is processed through interconnected networks of layers of computational elements (nodes) that emulate neural interactions in the human brain. During training, the model parameters are iteratively adjusted to detect and amplify significant and specific features within input data and optimize an objective function [22].

### 2.2 Systems for automatic medical image analysis

AI methods, which are typically characterized by a complex and very elaborate process to produce reliable results, can perform various tasks such as image classification, lesion segmentation and detection, and the discovery of new biological and prognostic biomarkers, facilitating the work of researchers in this analysis process.

**Fig. 2:**
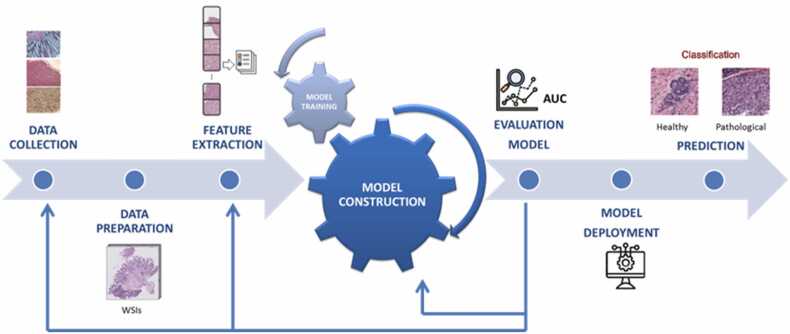
Automatic histological image analysis process: data collection, data preparation, features extraction, model construction and training, model testing and evaluation, model deployment, and image classification. During the construction of the model, an optimal artificial neural network is selected based on the training dataset and the problem to be solved (image classification, object detection, tissue recognition, etc.). The network learns from input data the right classification or prediction, and after the training phase, its performance is evaluated to test and validate the model itself. If the predictive outcomes are not satisfactory, the model should be further improved giving feedback in the "model construction and training" step which allows adjustments of some parameters/features. In some cases, it could be required to return to the "data collection" step to modify the data to be entered into the machine learning process.

In medicine, automated image analysis has enabled earlier detection of malignant lesions and prediction of the biology and progression of some tumors, with the potential to improve patient care, disease prevention, lower healthcare costs and enhance drug discovery. A typical automated image analysis process is described in Fig. 2.

Among the various AI techniques, DL and radiomics are particularly suitable for imaging and automated image analysis and are the most widely used methods for endoscopic and histological data analysis [23].

DL is inspired by human neural networks and implements artificial neural networks (ANNs), whose structure makes them particularly suitable for analyzing complex data. Such networks are made up of nodes (like neurons) distributed over several layers, each of which corresponds to features of the input or, more precisely, of the image to be analyzed (e.g. brightness, thickness, homogeneity, or other more abstract features) [24], which are suitably processed in subsequent layers until the final classification is obtained. Depending on the number of layers that make up the network, ANNs can be shallow or deep; the most used for medical images are convolutional neural networks (CNNs).

CNNs operate by subsampling the image into blocks and analyzing each block through filters/levels that detect the quantification of the features within them [25]. These networks can perform automated tasks on the input image, such as classification, object detection, segmentation, and other image generation tasks [26].

For DL networks, the training process can be supervised or unsupervised. In supervised training of an ANN, a set of labeled images is provided as input, already classified by an external human supervisor, allowing the network to recognize and learn the features associated with the input data, fine-tuning the weights of the network over subsequent iterations. To improve the performance of the network, especially in loosely supervised algorithms, attention constraint models can be added to focus on specific features in the data [27]. At the end of training, an optimal model capable of classifying new images with better performance is obtained.

Conversely, in unsupervised learning, unlabeled imaging data is provided, allowing the network to discover hidden relationships or features within the images without the predictive result being influenced by human supervision [28]. This type of learning has greater potential to uncover new patterns, relationships, and insights that may not have been previously identified, enabling a deeper understanding of the input data without providing given labels.

However, both supervised and unsupervised learning are affected by the "black box effect": the human brain cannot understand the thousands of parameters that ANNs use to extract models, and the researcher can't follow the evaluation of the model and thus explain it or identify problems in it. Radiomics is an interdisciplinary subfield of AI that uses various ML or DL approaches to extract information from medical images. For example, to analyze transversal images and capture features that are not easily perceived by the human eye, including the quantification of visual differences in image intensity, shape, consistency and spatial relationships [29].

Radiomics is generally used to analyze images obtained by MRI, CT, PET, and US. Models with more accurate predictive performance are achieved by combining radiomics with other clinical or omics data [23]. In the oncology field, these methods have identified signatures that can predict outcome, risk of distant metastasis and tumor biology [30].

In the field of IBD radiomics, analysis of IUS (intestinal ultrasound) images has accurately differentiated abnormal from normal images [31]. Furthermore, a radiomics diagnostic model based on multi-slice computed tomography (MSCT) and clinical factors has been developed to stratify fibrosis [32].

### 2.3 Artificial intelligence applications for histologic analysis in IBD

The advent of automated analysis of histopathological images was enabled by the digitization of whole slide images (WSIs) and the use of DL methods to extract underlying features or information from them. The usefulness of AI in prognostic models based on histopathological evaluation of IBD biopsies is still lacking. However, emerging studies describe its potential applications in some specific contexts [33], [34]. A summary of the major studies investigating AI models applied to histopathology in IBD is shown in Table 1.

**Table 1:** Summary of the key studies investigating AI models applied to histopathology in IBD.

	AI FOR HISTOLOGIC ANALYSIS IN IBD	AI Classifier	Populations	Primary outcomes/Clinical results	Performance					
					Precision	Sensitivity	Specificy	Accuracy	AUC	F1 score
	AI STUDIES IN IBD PEDIATRIC PATIENTS									
	Mossotto et al. [35]	Unsupervised model for initial clustering of dataset; supervised models: Boosted and Bagged Trees, linear discriminant analysis and SVMs	287 IBD patients: 178 CD, 80 UC and 29 IBDU	Classification of PIBD using endoscopic and histological data	For supervised models optimized during validation: 0.89 with endoscopic data; 0.81 with histological data; 0.91 with combined endoscopic/histological data	For supervised models optimized during validation: 0.68 with endoscopic data; 0.86 with histological data; 0.83 with combined endoscopic/histological data		During the training and the testing for the best model (SVM): 0.80; for supervised models optimized during validation: 0.71 with endoscopic data; 0.77 with histological data; 0.83 with combined endoscopic/histological data	For supervised models optimized during validation: 78% utilising endoscopic data; 82% with histological data; 82.7% with combined endoscopic/histological data; 87% with the best model with an independent cohort	For supervised models optimized during validation: 0.75 with endoscopic data; 0.83 with histological data; 0.87 with combined endoscopic/histological data
	Birimberg-Schwartz et al. [36]	CART	749 IBD patients: 236 CD, 272 UC and 241 IBDU	Classification of PIBD using endoscopic, histological and clinical data		During validation: 0.80 differentiating UC from CD and IBDU; 0.78 differentiating CD from IBDU and UC	During validation: 0.84 differentiating UC from CD and IBDU; 0.94 differentiating CD from IBDU and UC			
	Dhaliwal et al. [37]	SNF for initial clustering of dataset; RF	74 colonic IBD patients: 56 UC and 18 colonic CD	Classification of colonic PIBD using endoscopic, histological, radiological and clinical data				During the training: 0.97; during validation: 1		
	Liu et al. [38]	Different ML models: naive Bayes–based model, CatBoost, AdaBoost, RF, DT, Bagging, GradientBoosting, and LR	400 UC treatment-naive patients	Prediction of therapy response in pediatric UC patients using histomic features	For the best model (RF) during validation: 0.91	For the best model (RF) during validation: 0.84	For the best model (RF) during validation: 0.94	For the best model (RF) during the training with all 250 features: 0.92; for the best model (RF) during validation using 18 features: 0.90	For the best model (RF) during the training with all 250 features: 92%; for the best model (RF) during validation using the 18 features: 89%	For the best model (RF) during validation using the 18 features: 0.87
	AI STUDIES FOR DISEASE SEVERITY EVALUATION									
	Matalka et al. [39]	PNN	130 subjects: 87 IBD patients and 43 HC	Development of a novel automated system to recognize and classify architectural distortions of mucosal crypts	During the training: 1 for HC and all IBD grades; during the testing: 1 for HC, 0.92 for IBD grade 1, 0.9 for IBD grade 2, and 1 for IBD grade 3; overall system performance: 0.98	During the training: 1 for HC and all IBD grades; during the testing: 0.93 for HC, 0.92 for IBD grade 1, 1 for IBD grade 2, and 1 for IBD grade 3; overall system performance: 0.98				
	Pradhan et al. [40]	DL model (SegNet) and ML approach	20 multimodal images from 20 IBD patients	Assessment of IBD severity						For the best performance during validation: 0.75
	Peyrin-Biroulet et al. [41]	CNN modules and RF classifier module	200 histopathology slides from UC patients	Development of an automated histological scoring for histological disease activity in UC				The average of intraclass correlation coefficient (ICC) among the histopathologists: 89.3; the average ICC between histopathologists and the AI tool: 87.2		
	Klein et al. [42]	Multivariate regression models and neural network (NNET)	Colonic biopsies from 105 CD patients	Prediction of the clinical phenotype according to disease behavior and therapy decisions		During the testing for the NNET model: 0.81 to discriminate B1 and B2 phenotypes; 0.69 to discriminate B3 and B1 phenotypes; 0.64 to predict the need for surgery	During the testing for the NNET model: 0.74 to discriminate B1 and B2 phenotypes; 0.76 to discriminate B3 and B1 phenotypes; 0.72 to predict the need for surgery	During the training for the NNET model: 0.82 to discriminate B1 and B2 phenotypes; 0.7 to discriminate B1 and B3 phenotypes; 1 to predict the need for surgery; during the testing for the NNET model: 0.83 to discriminate B1 and B2 phenotypes; 0.78 to discriminate B1 and B3 phenotypes; 0.86 to predict the need for surgery	During the testing for the NNET model: 74% to discriminate B1 and B2 phenotypes; 78% to discriminate B3 and B1 phenotypes; 72% to predict the need for surgery	
	Kiyokawa et al. [43]	DL model (EfficientNet)	68 CD patients	Prediction of postoperative recurrence of CD	During the testing: 0.96	During the testing: 0.96		During the testing: 0.97	During the testing: 99%	During the testing: 0.96
										
	AI STUDIES BASED ON EOSINOPHILS COUNT AND NEUTROPHILS IDENTIFICATION									
	Niels Vande Casteele et al. [44]	CNN	88 UC patients	Identification of associations between eosinophil density, histological and clinical features in active UC	During the training: 0.92	During the training: 0.86		During the validation: ICC ranging from 0.805 to 0.917		During the training: 0.89
	Gui et al. [45]	CNN	Digitalized biopsies from 307 UC patients	Evaluation of inflammatory activity in UC patients to predict histological remission		For the neutrophil identification: 0.81 during validation and 0.80 during testing; for the UC activity prediction: 0.67 during validation and 0.78 during testing		For the neutrophil identification: 0.87 during validation and 0.89 during testing; for the UC activity prediction: 0.83 during validation and 0.86 during testing		For the neutrophil identification: 0.81 during validation and 0.84 during testing; for the UC activity prediction: 0.74 during validation and 0.82 during testing
	Iacucci et al. [46]	CNN	Digitalized biopsies from 273 UC patients	Distinction of active from quiescent UC and prediction of the endoscopic assessment and occurrence of flares at 12 months		For histologic remission/activity prediction: 0.89 during testing and 0.92 during validation	For histologic remission/activity prediction: 0.85 during testing and 0.81 during validation	For histologic remission/activity prediction: 0.87 during testing and 0.90 during validation	For histologic remission/activity prediction: 87% during testing and 90% during validation	Fo histologic remission/activity prediction: 0.84 during testing and 0.93 during validation
	Ohara et al. [47]	DL-based models	114 UC patients in endoscopic remission	Prediction of clinical relapse of UC	NA	NA	NA	NA	NA	NA
	Najdawi et al. [48]	CNN, RF	Left colon and rectum biopsies from 334 UC patients	Development of models to quantify histological features from WSI images of UC and evaluate disease activity				For histological remission classification: 0.97		
	Rymatczyk et al. [49]	RNN, FV + RF, and Self-Attention Attention-based Multi-instance Learning Pooling (SA-AbMILP)	Biopsies from 302 CD patients and from 887 UC patients	Prediction of disease assessment by exploiting four selected histological features				For the best model (SA-AbMILP): 0.87 to 0.94 for colon in both CD and UC; 0.76 to 0.83 for ileum CD		
	Furlanello et al. [50]	DL model (StarDist)	Public dataset of 4981 annotated images for training model and 356 intestinal biopsies of 24 CD, 19 UC, and 9 HC as external validation cohort	Development of an AI-based scoring system for quantifying basal plasmacytosis				0.90 for the assessment of basal plasmacytosis		
	AI STUDY FOR INFLAMMATION-ASSOCIATED DYSPLASIA									
	Noguchi et al. [51]	CNN	25,849 patches from WSIs of 12 patients with colitis-associated neoplasia	Prediction of p53 status from histological images	Ranging from 0.82 to 0.89	Ranging from 0.73 to 0.83	Ranging from 0.91 to 0.92	Ranging from 0.86 to 0.91		Ranging from 0.77 to 0.86

AI: Artificial intelligence; AUC: Area under the curve; IBD: Inflammatory bowel disease; CD: Crohn's disease; UC: Ulcerative colitis; HC: healthy controls; IBDU: IBD-unclassified; PIBD: Paediatric inflammatory bowel disease; ML: Machine learning; SVM: Support vector machine; CART: Classification and regression tree; SNF: Similarity network fusion; RF: Random forest; DT: Decision tree; LR: Logistic regression; PNN: Probabilistic neural network; ANN: Artificial neural network; CNN: Convolutional neural network; RNN: Recurrent neural network; FV: Fisher vector; WSI: Whole-slide image; ICC: Interclass correlation coefficients; NA: No available.

### 2.4 AI studies in IBD pediatric patients

Among the few application studies of AI in IBD using histological data the work of Mossotto et al. [35] stands out, aiming to provide an accurate diagnosis of IBD in pediatric patients. Different ML models were trained and tested to classify disease subtypes, and it was found that unsupervised models could not clearly distinguish between CD and UC. However, hierarchical clustering identified four novel subgroups characterized by different colonic involvement. Patient classification was performed using three supervised ML models, each applied to endoscopic data only, histological data only, and combined endoscopic/histological data. The three models achieved accuracies of 71.0%, 76.9%, and 82.7% respectively, and when the best classifier was tested on an independent cohort of 48 patients, its accuracy increased to 83.3%.

Birimberg-Schwartz et al. [36] conducted a study to derive and validate criteria to standardize the classification of different IBD subtypes in 749 enrolled children with IBD. They developed a diagnostic algorithm based on Classification and Regression Tree (CART) modeling that was able to differentiate UC from CD and IBDU (IBD-unclassified) with 80% sensitivity and 84% specificity, and CD from IBDU and UC with 78% sensitivity and 94% specificity. To discriminate between UC and CD phenotypes in 74 pediatric IBD patients, Dhaliwal et al. [37] developed an algorithm to discover specific patterns from endoscopic, radiological, and histological data at baseline. The authors first trained a random forest (RF) classifier on the full dataset, which correctly discriminated 97% of 58 patients and identified the 7 most important features for accurate classification. Of these, three were histological (granulomas, patchy crypt distortion, patchy chronic inflammation) and four were endoscopic (skip lesions, ≥5 small discrete ulcers in the colon, ileitis with mild caecum, and relative patchiness). They then validated the classifier using the selected 7 top features and tested it on 15 previously unseen patients, achieving 100% accuracy in classifying patients.

In this context, it is important to keep in mind that in pediatric patients (<6 years old), IBD-like histological damage (both UC and CD) may be underlined by a monogenic disease related to primary immunodeficiency [52].

Recently, Liu et al. [38] published a new approach aimed at achieving personalized treatment for patients with pediatric-onset ulcerative colitis. This approach integrates digital histopathology-based histomics features with ML algorithms to predict patient responses to certain therapies. By selecting a subset of 18 histomics features, their model achieved an AUROC of 0.89 and an accuracy of 0.90. While these results demonstrate the model's potential to accurately classify pediatric patients, it is imperative to address disparities and biases in ML models related to race, gender, and socioeconomic status for standardized application in clinical settings.

### 2.5 AI studies for disease severity evaluation

As mentioned above, when IBD is suspected, the pathologist needs to assess both abnormalities in mucosal architecture and inflammatory features in endoscopic sampling to assess disease severity. In this area, AI tools have been explored for their ability to grade histological lesions and evaluate disease severity. In 2013, Matalka et al. [39] developed a computerized system based on the training of an ANN that could detect and classify architectural distortions of mucosal crypts. They aimed to classify intestinal samples from 130 subjects, including 43 healthy controls (HC) and 87 IBD cases of varying severity. After digitizing the histological images, they were filtered and segmented to facilitate crypt localization. After the training phase, a probabilistic neural network (PNN) successfully classified specimens into four categories based on the degree of architectural distortion: normal, mild, moderate, and severe. During the training, the classifier achieved a recognition rate of 100%, and during the testing, it attained accuracies of 100%, 92.31%, 90%, and 100% for the classification of healthy tissue, IBD grade I, IBD grade II, IBD grade III, respectively, achieving an average accuracy of 98.31% and correctly diagnosing 116 out of 118 samples.

In 2019 Pradhan et al. [40] employed non-linear multimodal imaging to measure biomolecular changes in different tissue regions, such as the crypt and mucosal region, for real-time assessment of IBD severity. They used a DL model, called SegNet, to systematically segment the crypt and mucosa region and compared its performance with a classical ML approach based on a simple pixel classification problem. After training, the SegNet system achieved an overall F1 score (i.e. harmonic mean of precision and recall, a useful metric commonly adopted to classification models when there is an imbalance between classes) of 0.75 and outperformed the classical ML approach for crypt and mucosa segmentation.

More recently, to assess disease severity, Peyrin-Biroulet et al. [41] utilized an automated image analysis approach and ML algorithms on 200 histological images from UC patients to compare the performance of the AI system with the assessments of four independent histopathologists. The image dataset was divided into a training set and a test set, and manual (i.e. pathologist) and AI-automated measurements were evaluated using the intraclass correlation coefficient (ICC). Despite the small dataset, the authors found a high correlation among histopathologists and between histopathologists and the AI system, which was 89.33 and 87.20, respectively.

In 2017 Klein et al. [42] analyzed colonic biopsies from CD patients with at least five years of post-biopsy clinical follow-up using multivariate analysis and neural network models (NNET) to predict the clinical phenotype according to disease behaviour (B1 inflammatory, B2 stricturing, and B3 penetrating both internal and perianal) and to guide treatment decisions. The NNET model was able to discriminate B1 and B2 phenotypes (with an accuracy of 81.6% in the training set and 83.3% in the testing set) and to predict the need for surgery (with an accuracy of 100% in the training set and 86% in testing), while its performance decreased in the discrimination of B1 and B3 phenotypes (with an accuracy of 70% in the training set and 78% in testing).

Kiyokawa et al. [43] published the first study on DL analysis of histological images to predict postoperative CD recurrence and extract associated histological features. Their model analyzed 550 WSIs from surgical specimens of 68 patients and achieved high predictive accuracy with an AUROC of 0.995, suggesting the important role of adipocyte shrinkage and mast cell infiltration as important histological features associated with disease recurrence.

### 2.6 AI studies based on eosinophils count and neutrophil identification

Cellular quantification techniques can be used to detect specific histological alterations in biopsies, such as inflammatory cellular counts or qualifications (i.e. neutrophils vs eosinophils).

Recently, Niels Vande Casteele et al. [44] developed a DL algorithm to analyze WSIs of colon biopsies to identify associations between eosinophil density and histological and clinical features in active UC. Colon biopsies from 88 UC patients were digitized and used to manually annotate eosinophil counts by gastrointestinal pathologists. These data were used to train the model, which showed a sensitivity of 86.4%, an accuracy of 91.8%, and an F1 score of 0.89. During the validation phase, eosinophil counts from 20 different areas not used for training were analyzed, and the algorithm showed near-perfect agreement with manual eosinophil counts. The study found associations between eosinophil density, histological activity, and clinical characteristics, such as a correlation between eosinophil density and disease extent. In addition, significantly lower eosinophil density was observed in UC patients treated with corticosteroids.

Gui et al. [45] developed a simple histological index to assess inflammatory activity in UC patients, called the histological remission index (PHRI), and an AI diagnostic system capable of measuring it and predicting histological remission. The authors used histological slides from 138 UC biopsies representing different grades of inflammation to train and test a CNN model to detect neutrophils, calculate the PHRI, and identify disease activity by classifying WSIs into histological remission or non-remission. During the test and validation phases, the AI system achieved accuracies of 0.89 and 0.87 for neutrophil detection, respectively. Similar values were obtained for the classification of UC activity, with an accuracy of 0.86 during testing and 0.93 during validation. This study demonstrated that an AI algorithm based on neutrophil detection can accurately differentiate active from quiescent disease and predict histological remission. The same group then improved this model using a larger dataset of 535 UC biopsies and applied it to predict disease flare based on pre-specified clinical outcomes [46]. The CNN model predicted the presence of endoscopic inflammation in the biopsies with over 80% accuracy, and outcome stratification achieved similar results between the AI assessment and that of human pathologists.

In 2022, Ohara et al. [47] applied a quantitative method using a DL-based model to predict mucin depletion, a histological factor associated with clinical relapse in UC patients. Their model was able to measure goblet cell mucus and epithelial cells from WSIs of biopsy specimens with high accuracy and calculate their area ratio. This ratio was strongly correlated with mucin depletion, allowing for the prediction of clinical relapse.

Najdawi et al. [48] developed CNN models to quantify histological features of UC from WSI images and evaluate disease activity. The models performed tissue segmentation and cell classification, and these predictions were used to generate human-interpretable features, such as cell densities, cell count proportions, and tissue area proportions. The authors developed several models to identify neutrophils, plasma cells, intraepithelial lymphocytes, non-intraepithelial lymphocytes, eosinophils, enterocyte modification, and colonocyte muciparity, which showed strong correlations with disease severity and pathologist-assigned histological index scores. The CNN models were validated using the generated features as input to an RF classifier, which accurately predicted histological index scores on an independent test set and predicted histological remission based on the absence of neutrophil extravasation with a high accuracy of 0.97.

Similarly, Rymatczyk et al. [49] have developed DL models to predict disease assessment using the global histological activity score for CD and the Geboes histopathology score for UC. The best model, based on multiple instance learning and the attention mechanism, was able to discriminate between the presence and absence of pathology using four selected histological features (surface epithelium, crypts, infiltrate of lamina propria mononuclear cells, or neutrophils), with accuracies ranging from 87% to 94% for the colon in both CD and UC and from 76% to 83% for the ileum in CD.

More recently, Furlanello et al. [50] developed an AI-based scoring system to aid in the diagnosis of IBD by semi-automatically quantifying basal plasmacytosis. Their training dataset consisted of 4981 annotated images from a public dataset, while the validation cohort was characterized by 356 intestinal biopsies from patients with CD, UC, and HC. The authors developed a DL model, based on a previous algorithm called StarDist, for plasma cell segmentation that accurately replicated human assessment of basal plasmacytosis, a key feature in the differential diagnosis between IBD and non-IBD colitis, highlighting the value of AI models as a potential adjunct to IBD diagnosis.

### 2.7 AI study for inflammation-associated dysplasia

Patients with long-term UC have an increased risk of developing colorectal cancer (CRC), known as colitis-associated cancer (CAC). In these patients, histological evaluation plays a critical role, and immunohistochemistry for p53 along with hematoxylin and eosin staining is traditionally used to accurately diagnose these pathological conditions [53]. Noguchi et al. [51] developed a CNN model to predict p53 status from histological images. The model, trained on 80% of 25849 patches of whole slide images (remaining 10% for validation and 10% for final testing) categorized as p53 positive, p53 negative, and p53 null slides, achieved an accuracy ranging from 86% to 91%. This demonstrates that AI can be a more efficient and cost-effective method for detecting p53 immunohistochemical staining.

## 3 Discussion

Accurate diagnosis, particularly differentiating between IBD and non-IBD colitis and subclassification of IBD into UC and CD, is crucial for correct disease management, reducing the risk of long-term complications and improving the quality of life of patients. Over the past five years, AI, particularly ML and DL, has seen exponential growth in IBD research. These innovative approaches mimic human problem-solving, including decision-making processes and self-improvement, through algorithms that reveal patterns within datasets. ML, using thousands of computational iterations, can “learn” the unique signature of features (including images or data) associated with a given classification, including specific IBD subtypes. These advances have encouraged the application of AI to improve the diagnosis of IBD patients by facilitating the identification and classification of specific patterns and lesions in histological images, even at the microscopic level. AI can also predict treatment response based on diagnosis and detect subcellular and molecular changes that indicate disease severity or its progression over time.

In this review, we describe various applications of AI in the analysis of histological features on mucosal samples from patients with suspected IBD, including mucosal changes and inflammatory infiltrates and their severity assessment. The available studies highlight the promising results obtained to date and the current limitations that prevent the effective deployment of these methods as support tools. While literature data seem to confirm that AI can better detect lesions in endoscopic and histological settings, blinded data, i.e. AI histological report alone or AI endoscopic report alone, is not useful to define IBD diagnosis, as shown by Mossotto and colleagues [35]. They used a versatile approach with a wide range of ML models to obtain a comprehensive evaluation of the features of histological images and endoscopic data and achieved higher classification accuracy by combining all these data. However, the study lacked a clear delineation between IBD subtypes, and the unsupervised models failed to differentiate between CD and UC. Conversely, Dhaliwal et al. [37] achieved a classifier accuracy of 97% in distinguishing between disease subtypes using 58 IBD colonic pediatric patients with a 2:1 ratio of UC and CD, respectively. The authors reported that only 2 (1 CD and 1 UC) subjects were misclassified using the analyzed ML approaches. During the validation phase, they achieved a better performance (100%). The study suffers from several biases, including the unbalance of the subjects included (IBD colonic patients underwent colectomies, predominantly UC, and colonic CD patients with intact colon), randomization of the subjects, the use of a small validation dataset, simpler and more unbalanced (14UC and only one CD) that limits the generalizability of these results.

Concerning AI applications in the grading of architectural changes and inflammatory infiltrate, Matalka et al. [39] achieved high recognition accuracy with the PNN system, showing 100% accuracy during the training phase and high precision in the testing phase (an average accuracy of 98.31%). On the other hand, Pradhan et al. [40] had poorer performance, with an overall F1 score of 0.75 obtained by the SegNet system. These results highlight that despite the use of a multimodal imaging approach, which allowed for a more comprehensive assessment of histological and biomolecular features, further improvements are needed to increase the accuracy and reliability in identifying cryptic and mucosal regions. The review also focuses on studies in which AI algorithms have been developed to detect specific cell types, such as eosinophils and neutrophils, showing potential to predict treatment outcomes and disease activity. Niels Vande Casteele et al. [44] used an advanced DL approach for eosinophil quantification and demonstrated good performance in identifying eosinophils and assessing their density and associations with clinical and histological features.

Gui et al. [45] developed an AI diagnostic system capable of measuring a histological remission index to standardize the evaluation of inflammatory activity in UC and identify histological remission, thus facilitating objective assessment of treatment response. Subsequent refinement of the model made it the first AI tool able to predict and stratify the risk of relapse based on histological data, demonstrating a strong association between histological activity and disease relapse, independent of pathologist classification or CNN model. Future integration of histological and endoscopic AI models into a single tool is likely to improve these predictions. Finally, Najdawi's work [48] used a CNN to accurately classify histological features of IBD from WSI images, providing an advanced method for automated image analysis and disease severity assessment. However, the lack of detail in the description of the specific features identified by neural networks may hinder the understanding and interpretation of the results. The generalizability of the findings may also be limited by the size and representativeness of the sample used, which requires greater diversity in sample composition to ensure the validity and applicability of the findings to a broader IBD patient population. The studies discussed highlight the potential of AI to improve diagnostic and prognostic capabilities in IBD management through automated analysis of histological images. This approach can overcome human variability and biases in diagnostic processes, leading to more standardized assessments. AI is revolutionizing our understanding of the pathogenesis of several heterogeneous and complex diseases, hoping to improve the clinical classification of IBD subtypes. However, a notable lack of real-world clinical care application of AI in both CD and UC patients remains. After a thorough review of the current literature, it seems overly optimistic to expect that AI based on histopathological image studies will lead to significant advances in IBD, at least for now, as several challenges and biases remain to be still resolved. In fact, most studies report a validation score that is higher than the training score, or performance metrics that approach maximum values. This may indicate potential problems during the training process and may be due to several reasons, such as overfitting and underfitting biases, or bias-variance trade-offs. The main limitation of AI approaches remains the quality of the input data and adequate sample size, since the optimization of algorithms that mimic human problem-solving capabilities relies heavily on the robustness of the data on which the models are trained. Indeed, many studies suffer from distribution biases including selection, annotation, and unrepresentativeness of the data. Great attention needs to be paid to the selection and annotation of datasets, which should reflect the broad spectrum of patients with heterogeneous backgrounds and presenting factors, and to the development of simple and standardized procedures for data acquisition and management while preserving patient privacy and security.

In addition, researchers must play a key role in the effective selection and training of models to avoid methodology selection biases. They need to amend AI tools capable of providing reliable and certain predictions that can influence diagnostic and, consequently, therapeutic decisions for patients; to this end, multimodal and integrative approaches must also be promoted to combine histological information with a wide and diverse range of data to obtain increasingly complete and accurate information.

## 4 Conclusion

The intricate process of IBD diagnosis and differentiation could be revolutionized by AI, given its ability to accurately identify microscopic and sometimes latent disease features, predict histological remission, anticipate flare-ups and potential complications, and improve therapeutic management. However, several challenges remain and further improvements in the use of these methods are needed. We believe that in the next few years, thanks to international data-sharing initiatives, AI tools will be trained on complete images to reduce selection bias in the field. These tools will also be trained on large, unbiased datasets that accurately reflect the heterogeneity of IBD patient characteristics. The use of validated and standardized methods will improve histopathology workflows, enhancing their ability to distinguish different classes of IBD and distinguish them from non-IBD conditions.

## Author statement

I declare that this manuscript is original, has not been published before and is not currently being considered for publication elsewhere. I confirm that the manuscript has been read and approved by all named authors and that there are no other persons who satisfied the criteria for authorship but are not listed. I confirm that the order of authors listed in the manuscript has been approved by all the authors. All the authors understand that the Corresponding Author is the sole contact for the Editorial process and it is responsible for communicating with the other authors about progress, submissions of revisions and final approval of proofs.

## Funding

The study was funded by the “Ricerca Corrente” 2022–2024 and funding program of the Italian Ministry of Health and the Italian Next Generation EU Program (PNRR) PNRR-MAD-2022-12375729.

## CRediT authorship contribution statement

**Anna Lucia Cannarozzi:** Writing – review & editing, Writing – original draft, Methodology, Investigation, Formal analysis, Data curation, Conceptualization. **Luca Massimino:** Visualization, Investigation, Formal analysis. **Silvio Danese:** Writing – original draft, Visualization, Resources. **Orazio Palmieri:** Writing – review & editing, Writing – original draft, Visualization, Supervision, Resources, Project administration, Methodology, Investigation, Funding acquisition, Formal analysis, Data curation, Conceptualization. **Francesco Perri:** Writing – original draft, Investigation, Data curation. **Federica Ungaro:** Writing – original draft, Visualization, Investigation, Data curation. **Giuseppe Biscaglia:** Writing – original draft, Validation, Data curation. **Fabrizio Bossa:** Writing – review & editing, Writing – original draft, Methodology, Conceptualization. **Anna Laura Di Brina:** Visualization, Methodology, Data curation. **Francesco Giuliani:** Visualization, Methodology. **Anna Latiano:** Writing – review & editing, Visualization, Validation, Formal analysis, Data curation. **Tommaso Lorenzo Parigi:** Writing – review & editing, Formal analysis.

## Declaration of Competing Interest

S Danese has served as a speaker, consultant, and advisory board member for Schering-Plough, AbbVie, Actelion, Alphawasserman, AstraZeneca, Cellerix, Cosmo Pharmaceuticals, Ferring, Genentech, Grunenthal, Johnson and Johnson, millennium Takeda, MSD, Nikkiso Europe GmbH, Novo Nordisk, Nycomed, Pfizer, Pharmacosmos, UCB Pharma and Vifor. The other authors have no relevant disclosures.
